# Variability in *CitXET* expression and XET activity in *Citrus* cultivar *Huangguogan* seedlings with differed degrees of etiolation

**DOI:** 10.1371/journal.pone.0178973

**Published:** 2017-06-15

**Authors:** Bo Xiong, Xianjie Gu, Xia Qiu, Zhixiang Dong, Shuang Ye, Guochao Sun, Shengjia Huang, Xinya Liu, Lijuan Xi, Zhihui Wang

**Affiliations:** 1 College of Horticulture, Sichuan Agricultural University, Chengdu, Sichuan, China; 2 Mianyang Academy of Agricultural Sciences, Mianyang, Sichuan, China; 3 Institute of Pomology and Olericulture, Sichuan Agricultural University, Chengdu, Sichuan, China; Universidade de Lisboa Instituto Superior de Agronomia, PORTUGAL

## Abstract

Considering the known effects of xyloglucan endotransglycosylase (XET) on plant growth and development, we aimed to determine whether XETs help to regulate the growth and elongation of *Huangguogan* shoots and roots. We confirmed a possible role for XET during seedling etiolation. Our results revealed that the roots of etiolated seedlings (H-E) were longer than those of green seedlings (H-G). However, shoot length exhibited the opposite pattern. We also observed positive and negative effects on the xyloglucan-degrading activity of XET in the root sub-apical region and shoots of etiolated *Huangguogan* seedling, respectively. There was a significant down-regulation in *CitXET* expression in the etiolated shoots at 15 days after seed germination. On the contrary, it was significantly increased in the root sub-apical region of etiolated and multicolored seedlings at 15 days after seed germination. The XET coding sequence (i.e., *CitXET*) was cloned from *Huangguogan* seedlings using gene-specific primers. The encoded amino acid sequence was predicted by using bioinformatics-based methods. The 990-bp *CitXET* gene was highly homologous to other *XET* genes. The CitXET protein was predicted to contain 319 amino acids, with a molecular mass of 37.45 kDa and an isoelectric point of 9.05. The predicted molecular formula was C_1724_H_2548_N_448_O_466_S_14_, and the resulting protein included only one transmembrane structure. The CitXET secondary structure consisted of four main structures (i.e., 21% α-helix, 30.72% extended strand, 9.09% β-turn, and 39.18% random coil). Analyses involving the NCBI Conserved Domains Database (NCBI-CDD), InterPro, and ScanProsite revealed that CitXET was a member of the glycosyl hydrolase family 16 (GH16), and included the DEIDFEFLG motif. Our results indicate that the differed degrees of etiolation influenced the *CitXET* expression pattern and XET activity in *Huangguogan* seedlings. The differential changes in XET activity and *CitXET* expression levels in *Huangguogan* seedlings may influence the regulation of root and shoot development, and may be important for seedling etiolation.

## Introduction

The genus *Citrus* of the family Rutaceae includes commercially important and widely cultivated fruit species [1]. In the same seed germination and seedling development conditions, *Citrus* cultivar *Huangguogan* plants produce a few etiolated seedlings. Etiolated, multicoloured, and green seedlings appeared on the fifth day after seed germination. The leaves of etiolated seedlings do not turn green even at 20 days after seed germination, and even death after further 10 days. In previous study, we found that etiolation decreased the leaf area and reduced the optical area, resulting in dwarf plants and weakening growth potential [2]. Etiolation, which is common in angiosperms, is a phenomenon that leaves are yellow when they grow in darkness. After seed germinating in darkness, seedlings undergo etiolated growth (i.e., skotomorphogenesis), and leaf color is dependent on carotenoids. This developmental step is characterized by a rapid elongation of the hypocotyl topped by a hook with underdeveloped cotyledons [3]. Etiolation decreases the leaf area, causes dwarfism in plants, lowers the growth potential, and may even cause to death. Over the past two decades, the growth and development of etiolated plants have been studied in terms of light regulation [4], endogenous abscisic acid [3], ethylene responses [5], phospholipid hydroperoxide glutathione peroxidase [6], riboflavin biosynthesis [7], and the proteome [8].

Xyloglucan endotransglucosylase/hydrolases (XTHs), which belong to glycosyl hydrolase family 16 (GH16), exhibit the activities of xyloglucan endotransglycosylase (XET) and xyloglucan endohydrolase (XEH) [9]. The XET and XEH activities occur throughout the growing tissues of monocots and dicots, suggesting that these enzymes are essential for plant development [10–12]. These enzymes have important roles during plant growth and differentiation [13,14] because they are directly involved in the initial assembly [15] and subsequent re-structuring [16] of the primary plant cell walls [17,18]. These enzymes are confirmed to function as XETs and/or XEHs [19]. Initially, XETs release a smaller xyloglucan from the reducing end of a donor xyloglucan, subsequently another xyloglucan chain is added to the newly generated free end [18,20,21]. The XETs, which lack hydrolase activity, have been identified in some charophytic algae and in all land plants [11,12,22]. They are considered to be involved in the molecular grafting or modification of the plant cell wall, but not in the breakdown of xyloglucans [20]. A few XTHs function primarily as XEHs [23].

The XET activity and *XTH* gene expression levels are correlated with cell expansion [10,24]. The considerable evidence that XTHs can serve as cell growth promoters is based on the results of molecular studies involving loss and gain of function [25–28]. The XTHs are usually encoded by a large multigene family. For example, there are 29 XTH genes in rice (*Oryza sativa*) [29], 41 in poplar (*Populus* spp.) [30], 22 in barley (*Hordeum vulgare*) [9], 25 in tomato (*Solanum lycopersicum*) [31], and 33 in *Arabidopsis thaliana* [32]. One-third of these genes are the result of genome duplications [33]. The XTHs are the main enzymes mediating plant cell wall restruction. Additionally, the correlation between *XTH* gene expression levels and cell expansion and morphology suggests that these enzymes play a key role in stress responses [34]. Microarray results have revealed that the *XTH* gene is differentially expressed in the roots and shoots of *A*. *thaliana* plants subjected to a 24-h drought stress treatment [35]. In well-defined topological regions of plants, the spatial regulation of *XTH* gene expression is helpful for strengthening or loosening the cell wall, which contributes to dehydration tolerance [34]. In angiosperms, the XTHs are associated with cell wall biosynthesis and degradation during seedling development [36–38]. In response to decreased exposure to blue or red light, these enzymes regulate petiole elongation [39,40].

Considering the known effects of XETs on plant growth and development, we aimed to determine whether XETs help to regulate the growth and elongation of *Huangguogan* shoots and roots. Another objective was to elucidate the *XET* gene expression pattern and function in etiolated seedlings. Thus, we identified and isolated the *Huangguogan XET* gene and completed bioinformatics-based analyses. We herein describe the growth of *Huangguogan* seedlings with differed degree of etiolation, and discuss its role during the elongation of the roots and shoots of etiolated seedlings. Our findings may be useful for characterizing the function of *CitXET* in *Huangguogan* root and shoot development during the process of etiolation.

## Materials and methods

### Plant materials

*Huangguogan* seeds were obtained from the Institute of Pomology and Olericulture, Sichuan Agricultural University, China. The seeds were presoaked in water for 4 h, incubated at 25 ± 1°C for 3 days, and then transferred to pots filled with vermiculite and perlite (1:1, v/v). The pots were placed in a growth chamber set at 25 ± 1°C and 50–60% relative humidity. The seedlings were exposed to a 12-h light/12-h dark photoperiod, and watered every 2 days. The etiolated (H-E), multicolored (H-M), and green (H-G) seedlings were harvested at 5, 10, 15, and 20 days after seed germination (i.e., emergence of the radicle through the seed coat). The collected samples were immediately frozen in liquid nitrogen and stored at −80°C.

### Root and shoot dry weight and length

Eight H-E, H-M, and H-G *Huangguogan* seedlings were collected at 20 days after germinating, and then divided into shoots and roots. The root and shoot length was measured using a vernier caliper. The shoots and roots were dried at 70°C for 24 h. The root and shoot dry weight was measured by an electronic balance.

### Enzyme extraction

For estimating enzyme activities, total proteins were extracted from the shoots and root sub-apical regions of the H-E, H-M, and H-G seedlings at different time points (i.e., 5, 10, 15, and 20 days after seed germination) as previously described [19,41].

### Quantitative real-time polymerase chain reaction analysis

Total RNA was extracted from the root sub-apical regions (10 mm to 50 mm distance from root cap) and shoots of H-E, H-M, and H-G seedlings using RNAiso Plus (TaKaRa, Dalian, China). First-strand cDNA was synthesized with the PrimeScript RT reagent Kit with gDNA Eraser (Takara, Dalian, China). To analyze the highly conserved *XET* gene, we aligned the following sequences, which were obtained from the NCBI database: *A*. *thaliana* (X92975.1), *Actinidia deliciosa* (L46792.1), *Vitis vinifera* (AY043238.1), *Solanum lycopersicum* (D16456.1), *Fragaria chiloensis* (GQ280283.1), *Pyrus pyrifolia* (EU432411.1), and *Malus domestica* (AY144593.1). We also searched the Citrus Genome Database (http://citrus.hzau.edu.cn) to identify the homologous citrus *XET* gene (*CitXET*). The Primer 3.0 online tool (http://bioinfo.ut.ee/primer3-0.4.0/) was used to design *CitXET*-specific primers (i.e., *CitXET*-F: 5′-ATGACGAATATACGTTTTTCATTT-3′ and *CitXET*-R: 5′-TCATATGTCTCTGTCTCTT CTGCAT-3′). These two primers along with those specific for *Actin* (GenBank: XM 006480741.2) (i.e., *Actin*-F: 5′-CCTCACTGAAGCACCACTCA-3′ and *Actin*-R: 5′-GTGGAAGAGCATACCCCTCA-3′) were synthesized by Sangon Biotech, China. The quantitative real-time polymerase chain reaction (qRT-PCR) experiment was conducted using SYBR Premix Ex Taq II (Takara, Dalian, China) and the CFX96 Real-Time PCR system (Bio-Rad, USA). The qRT-PCR experiment was completed using three separate biological replicates. The relative gene expression levels were calculated based on the 2^−ΔΔCT^ method, with a citrus *Actin* gene serving as the internal control.

### Cloning and sequence analysis of *CitXET*

Total RNA was extracted from *Huangguogan* as previously described [42]. First-strand cDNA was synthesized with the HiScript 1st Strand cDNA Synthesis Kit (Vazyme Biotech Co., Ltd, Nanjing, China). The extracted RNA was treated with DNase I (Invitrogen) to eliminate contaminating genomic DNA, and then stored at −20°C. The PCR amplification of the target sequence was completed in a 25-μL solution that included 12.5 μL 2× Taq Master Mix (Vazyme Biotech Co., Ltd, Nanjing, China), 1 μL forward and reverse gene-specific primers, 1 μL cDNA template, and double-distilled H_2_O up to 25 μL. The PCR program was as follows: 94°C for 3 min; 35 cycles of 94°C for 30 s, 55°C for 30 s, and 72°C for 90 s; 72°C for 5 min. The amplified target fragment was analyzed by agarose gel electrophoresis, and then purified using the Agarose Gel DNA Recovery Kit (TIANGEN Biotech Co., Ltd, Beijing, China). The target fragment was incorporated into the pMD19-T vector, which was then inserted into *Escherichia coli* DH5α cells and sequenced.

### Bioinformatics analysis

The BlastN online tool (https://blast.ncbi.nlm.nih.gov/Blast.cgi) was used to analyze the homology between *CitXET* and the other plant *XET* genes. The amino acid sequence encoded by *CitXET* was determined using the DNAMAN program. The amino acid composition, isoelectric point and molecular mass of the CitXET protein were calculated with the ExPASy ProtParam tool (http://web.expasy.org/protparam/). Additionally, we analyzed the protein transmembrane region with the TMHMM Server v. 2.0 (http://www.cbs.dtu.dk/services/TMHMM/), while the protein signal peptide was predicted using SignalP 4.1. Furthermore, SOPMA, ClustalX 1.83, BioEdit, and MEGA 7.0.12 program were used to compare the amino acid sequences and construct a phylogenetic tree. ESPript 3.0 was used for multiple sequence alignment and homology modeling. The secondary and tertiary protein structures were predicted using RCSB PDB and SWISS-MODEL [43].

### Statistical analysis

The data was analyzed using Duncan’s multiple range test in the XLSTAT program (version 2010) (*P* = 0.05 level of significance).

## Results

### *Huangguogan* seedling growth

**Fig 1:**
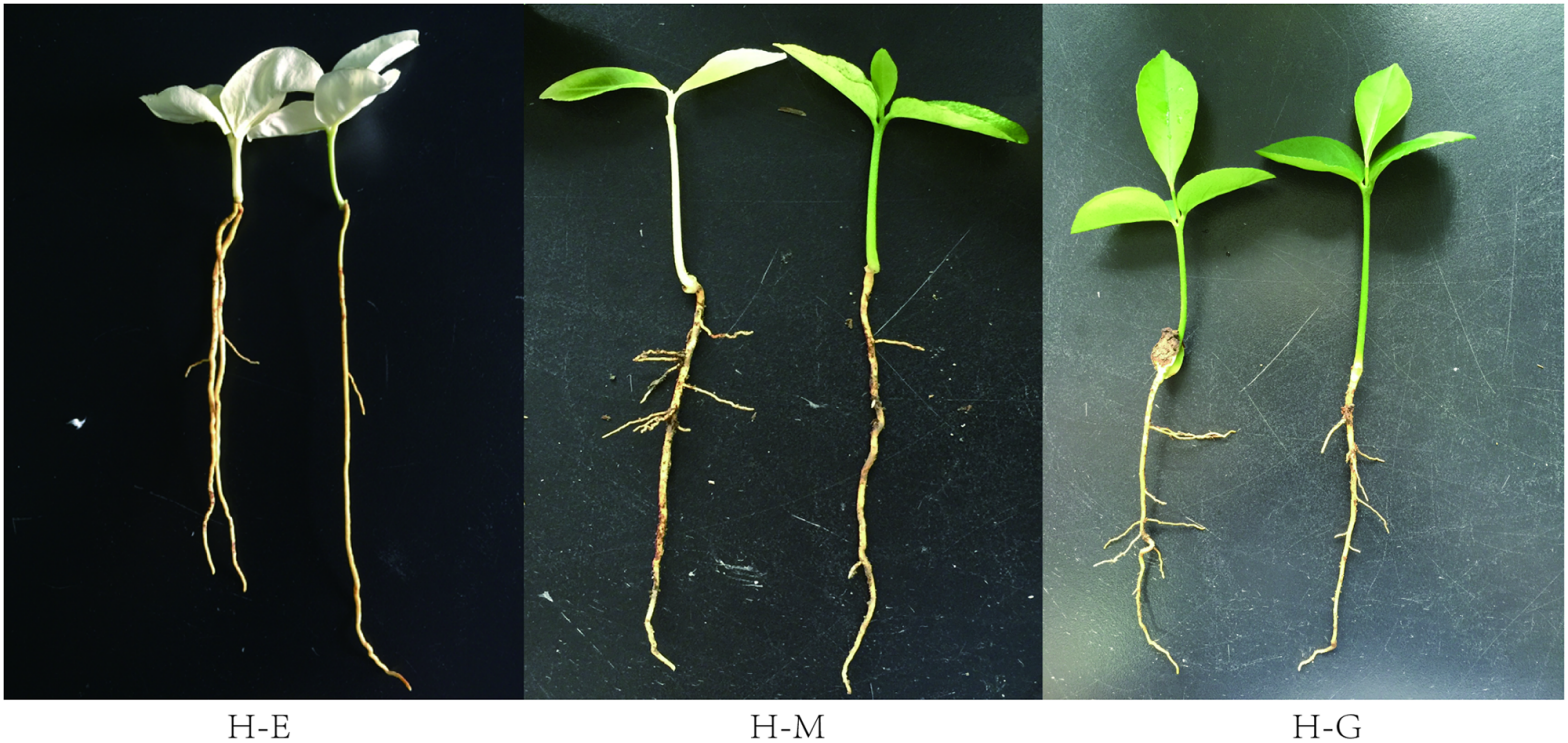
Figures of *Huangguogan* seedlings in 20 days after seed germination. H-E, etiolated seedlings. H-M, multicoloured seedlings. H-G, green seedlings.

The dry matter content as well as the root and shoot lengths of *Huangguogan* seedlings were measured at 20 days after seed germination (Fig 1). The dry weight, shoot length, and root-to-shoot ratio of H-E seedlings were significantly lower than those of H-G seedlings. However, the opposite trend was observed for root length (Table 1). These results suggest that the H-E seedling roots and shoots grow faster and slower than those of the H-G seedlings, respectively.

**Table 1:** Effect of etiolation on *Huangguogan* seedling growth.

Seedlings	Dry weight/g	Dry weight of Shoot/g	Dry weight of Root/g	Root length/cm	Shoot length/cm	Root shoot ratio
H-E	0.74±0.005c	0.25±0.009b	0.49±0.006c	21.35±0.71ab	3.64±0.04b	0.51±0.06b
H-M	0.83±0.009b	0.27±0.014b	0.56±0.008b	21.00±0.68bc	4.61±0.07a	0.48±0.05c
H-G	1.09±0.013a	0.42±0.018a	0.67±0.010a	19.01±0.63c	4.59±0.06a	0.63±0.08a

H-E, *Huangguogan* etiolated seedlings; H-M, *Huangguogan* multicolored seedlings; H-G, *Huangguogan* green seedlings. Different letters in each column indicate significantly different values (at *P* = 0.05 level).

### Identification and isolation of the *CitXET* gene

The cDNA produced by reverse transcription was used as the template for PCR amplifications. The 990-bp amplicons observed during agarose gel electrophoresis was consistent with the expected fragment size (S1 Fig). The results of the sequencing by GENEWIZ Biotechnology Co., Ltd. indicated that the amplified DNA fragment consisted of 990 bp. A comparison with other sequences using the Blastn and GenBank online tools revealed that *CitXET* was 99% homologous to the corresponding *Citrus sinensis* gene. Additionally, *CitXET* was 82%, 81%, 80%, and 82% homologous to sequences of *V*. *vinifera* (AY043237.1), *M*. *domestica* (EU494960.1), *P*. *pyrifolia* (EU432411.1), and *Glycine max* (NM_001253317.2), respectively. These results confirmed that the cloned sequence represented the *Huangguogan XET* gene.

**Fig 2:**
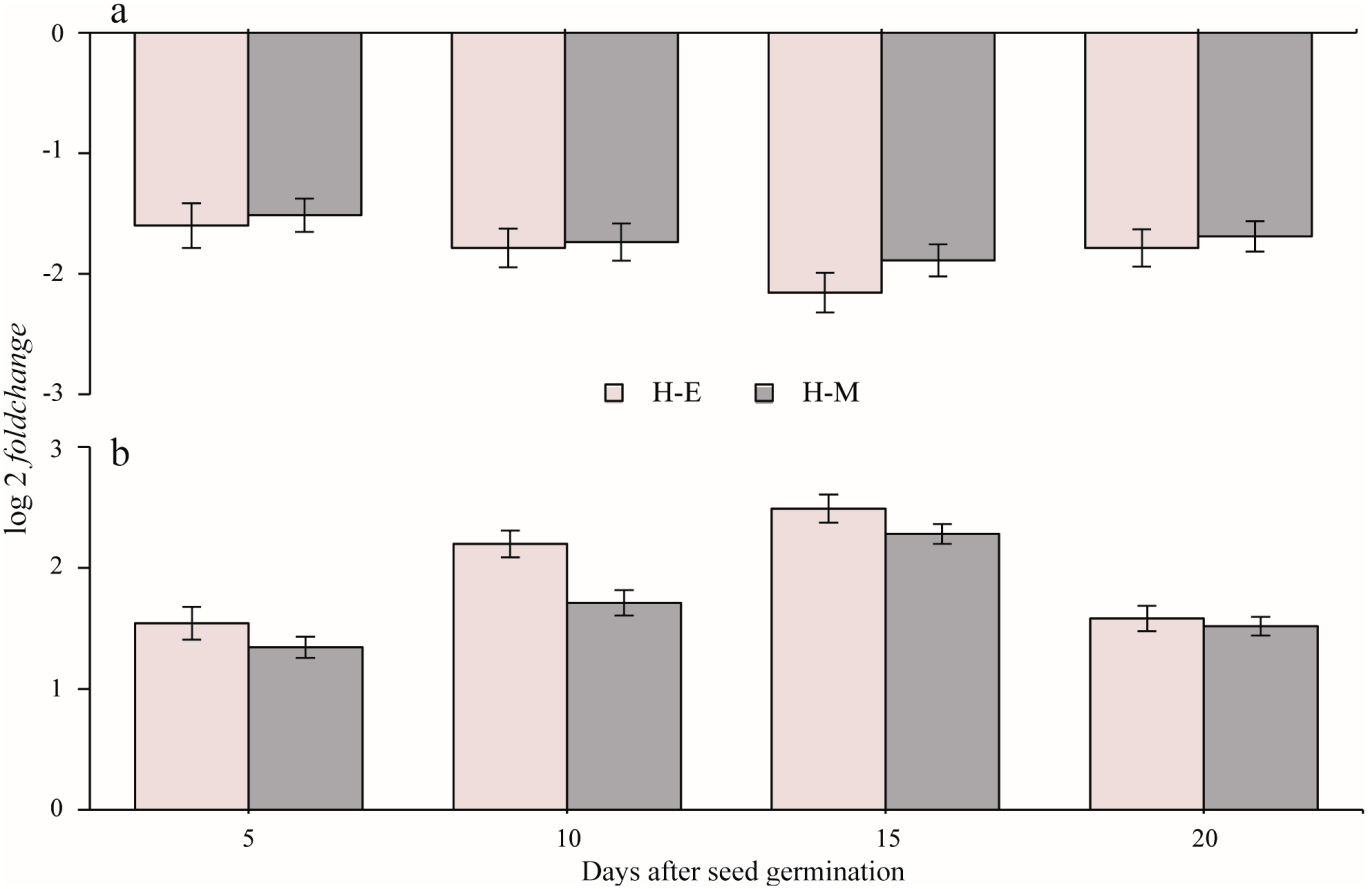
*CitXET* expression profiles of the etiolated (H-E) and multicolored (H-M) *Huangguogan* seedlings at different time points (i.e., 5, 10, 15, and 20 days after seed germination). **a**: shoots; **b**: root sub-apical region. The *CitXET* expression levels are provided as the transcript inhibition levels (log2 fold change) relative to the values for the green *Huangguogan* seedlings. Data are presented as the mean ± standard deviation of three independent replicates (*n* = 3).

We observed a slight but significant down-regulation in *CitXET* expression in the H-E shoots (log2 fold change = −2.15) at 15 days after seed germination (Fig 2a). For the other time points (i.e., 5, 10, and 20 days after seed germination), there was a small but consistent decrease in the *CitXET* expression levels of H-E and H-M seedlings (log2 fold change between −2 and −1). However, this decrease was considered insignificant. In the root sub-apical region (Fig 2b), a small but consistent increase in the *CitXET* expression levels of H-E and H-M seedlings was observed at 15 days after seed germination. The log2 fold change values for the *CitXET* expression levels in H-E and H-M seedlings were about 2.49 and 2.28 (i.e., up-regulated), respectively. At 10 days after seed germination, a significant up-regulation in *CitXET* expression was detected in H-E seedlings (log2 fold change = 2.20).

**Fig 3:**
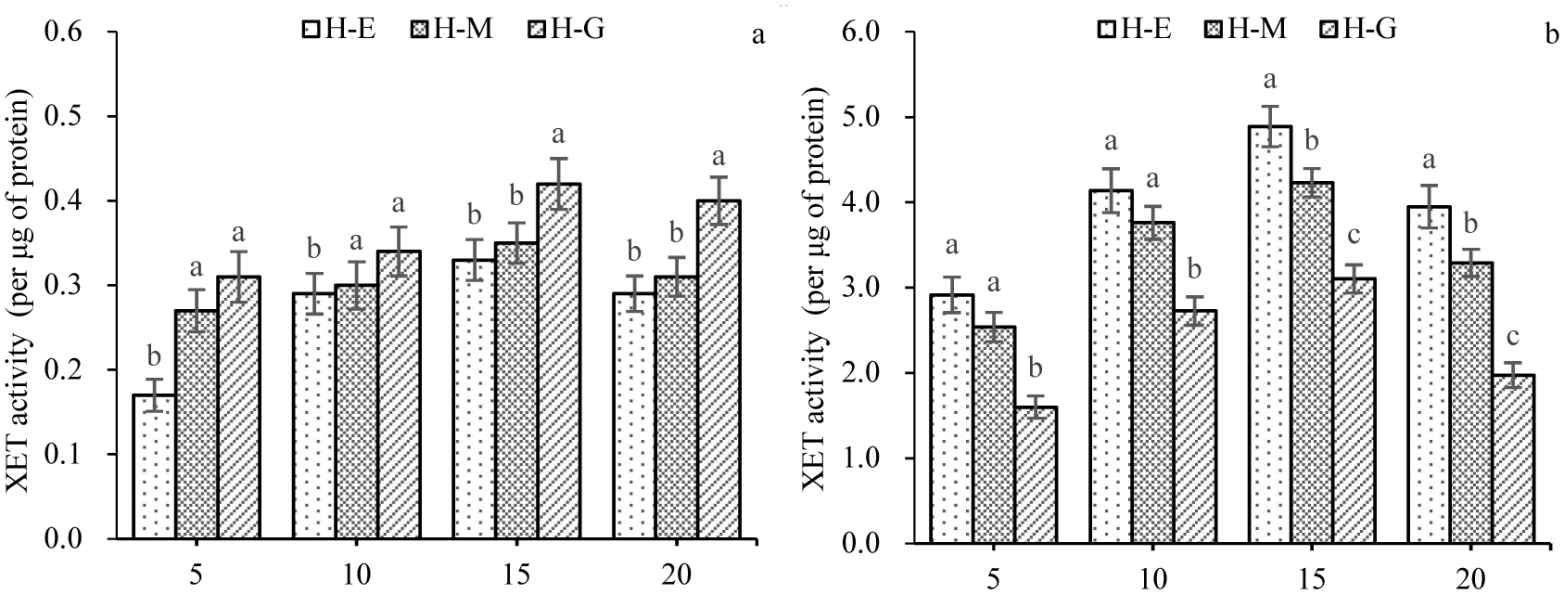
XET activity in excised segments of the etiolated (H-E), multicolored (H-M), and green (H-G) *Huangguogan* seedlings at different time points (i.e., 5, 10, 15, and 20 days after seed germination). **a**: shoots; **b**: root sub-apical region. Total proteins were extracted from seedlings, and XET activity was estimated using xyloglucan polymer as the specific substrate (extracted from tamarind flour). The data are presented as the mean of six biological replicates ± standard error. Duncan’s multiple range tests were used to establish statistical significance. Different letters indicate significantly different values (at *P* = 0.05 level).

The xyloglucan-degrading activity of XET during *Huangguogan* seedling etiolation was estimated using an iodine based detection of xyloglucans. Compared with H-G, a significant (*P* < 0.05) decrease in extractable enzyme activity of H-E was evidenced in the shoots for 5, 10, 15, and 20 days after seed germination. In shoots (Fig 3a), the basal levels for H-E (0.17 U/mg) and H-M (0.27 U/mg) seedlings were observed on day 5. The XET activities of root sub-apical region were slightly more active (>1.50 U/mg) than that of shoots. Gradual increases in activity were observed for H-E (0.29–0.33 U/mg), H-M (0.30–0.35 U/mg), and H-G (0.34–0.42 U/mg) seedlings between days 10 and 20 (Fig 3a). Maximum activity was recorded on day 15 in shoots and root sub-apical region. In shoots, XET activity of H-G seedlings was the most active, followed by that of H-M seedlings and H-E seedlings. But it was the opposite to the root sub-apical region, the XET activity of H-E seedlings was significantly (*P* < 0.05) higher than that of H-G seedlings (Fig 3b).

### Analysis of the *CitXET* sequence

**Fig 4:**
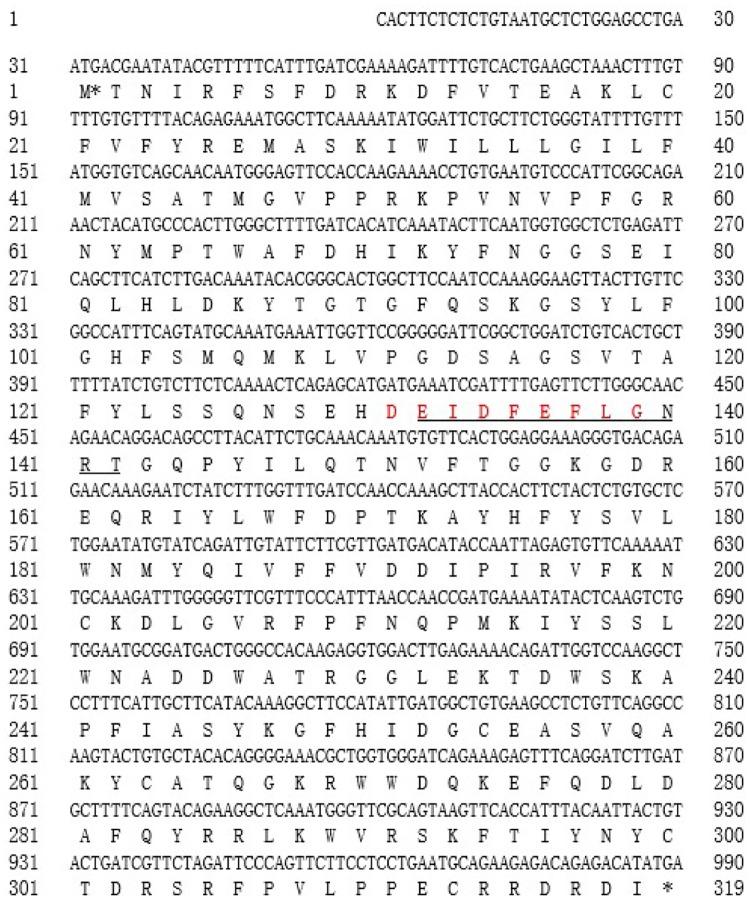
Deduced *CitXET* gene and encoded amino acid sequences. Underlined EIDFEFLGNRT was the conserved consensus signature motif of glycosyl hydrolase family 16 protein. The functional site (-DEIDFEFLG-) of most XTHs in family GH16 was highlighted in red color.

A 990-bp amplicon was generated using *CitXET*-specific primers. The encoded amino acid sequence was determined using the DNAMAN program. A subsequent search using the NCBI ORF finder and NCBI Protein-Blast algorithm revealed that *CitXET* containing a 960-bp coding region encoded a protein, which consisted of 319 amino acids (Fig 4). The ExPASy ProtParam tool indicated that the 319 CitXET amino acids formed a 37.45-kDa protein (molecular formula: C_1724_H_2548_N_448_O_466_S_14_), with an isoelectric point of 9.05. The most common amino acid was phenylalanine (29, 9.1%), followed by aspartic acid (22, 6.9%), glycine (22, 6.9%), and lysine (21, 6.6%). The least common amino acids were methionine (8, 2.5%), cysteine (6, 1.9%), and histidine (6, 1.9%). The instability index was calculated as 44.12. Additionally, the grand average of hydropathicity value was −0.398. We submitted the full-length *CitXET* sequence to the GenBank database using the BankIt tool (Accession number: KY576851).

### Secondary and tertiary protein structures of CitXET

**Fig 5:**
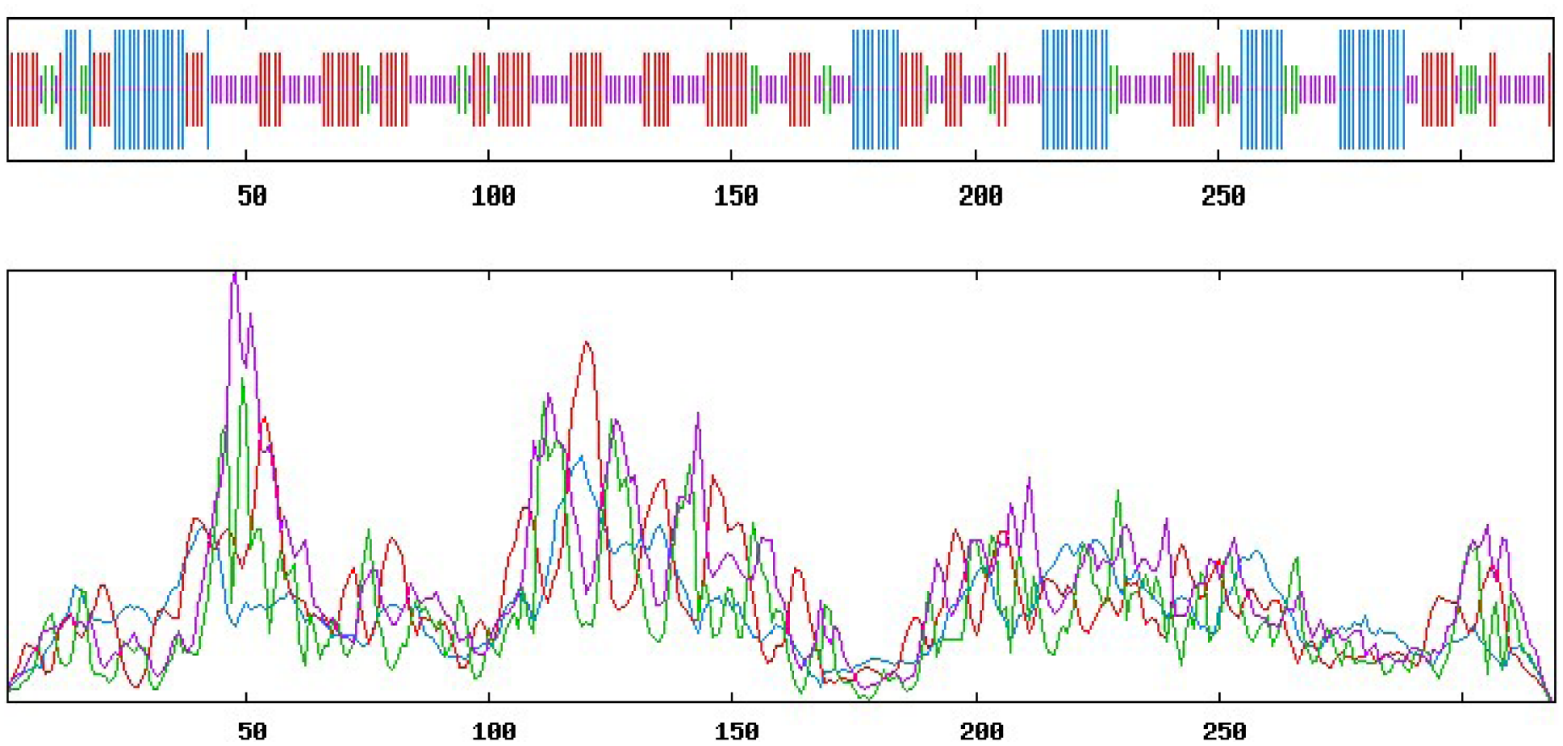
Predicted CitXET secondary structures. Blue line, α-helix; Red line, extended chain; Green line, β-sheet; Purple line, random curl.

**Fig 6:**
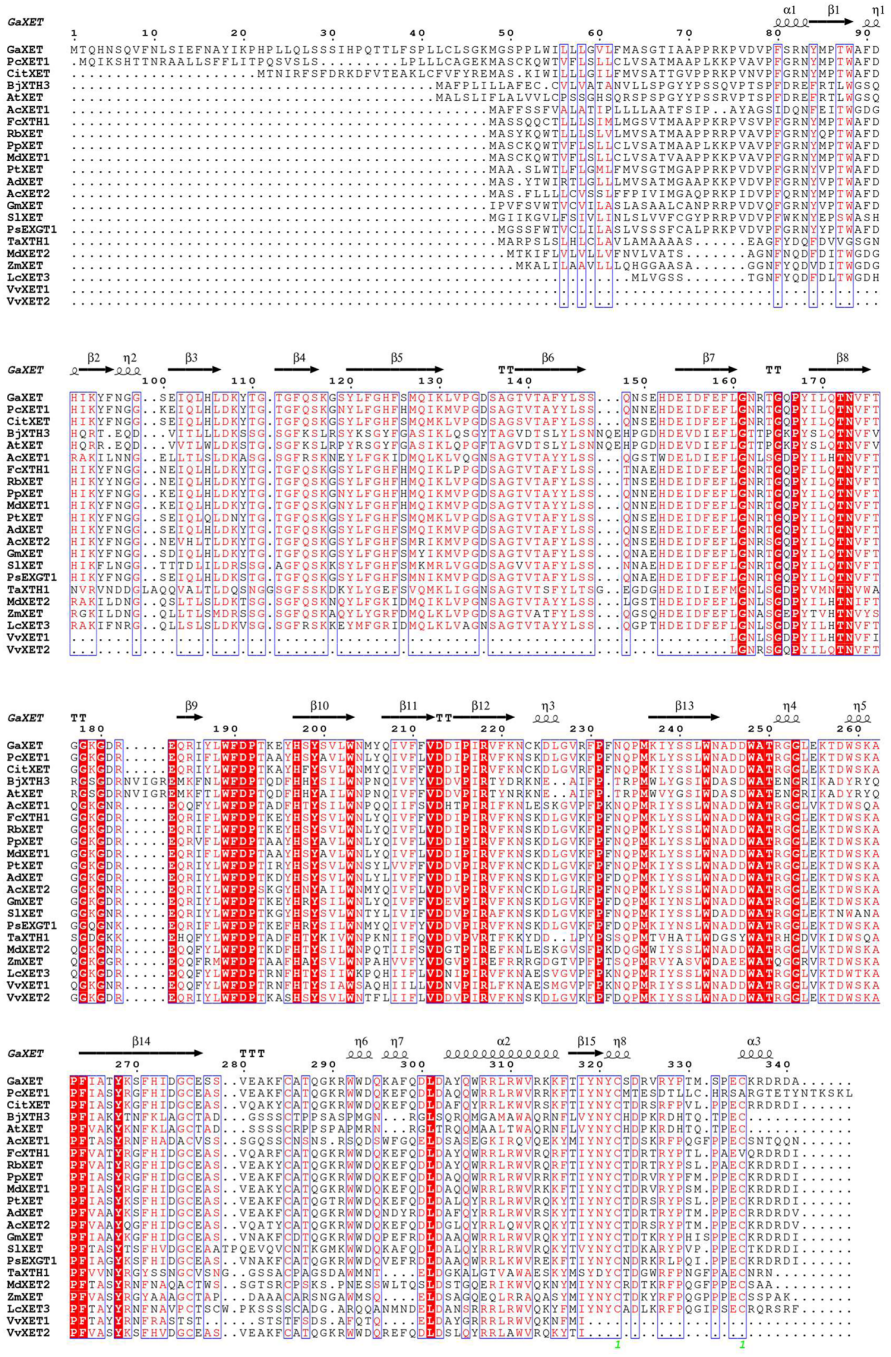
Multiple sequence alignment and homology modeling of XETs from *Huangguogan* and other plant species. Multiple alignment analysis of CitXET protein sequence was generated with the protein sequences of other known plant *XET* sequences from the NCBI database (https://www.ncbi.nlm.nih.gov/). *GaXET* (*Gossypium arboretum*, KHG12145.1), *PtXET* (*Populus trichocarpa*, XP-002297895.1), *AdXET* (*Actinidia deliciosa*, AAC09388.1), *VvXET2* (*Vitis vinifera*, AAK81881.1), *SIXET* (*Solanum lycopersicum*, BAA03923.1), *FcXTH1* (*Fragaria chiloensis*, ADE42488.1), *RbXET* (*Rosa x borboniana*, ABB86296.1), *PpXET* (*Pyrus pyrifolia*, ACA02823.1), *PcXET1* (*Pyrus communis*, BAC58038.1), *MdXET1* (*Malus domestica*, AAN07897.1), *GmXET* (*Glycine max*, BAA03922.1), *PsEXGT1* (*Pisum sativum*, BAA34946.1), *AcXET2* (*Annona cherimola*, ACK36946.1), *AcXET1* (*Annona cherimola*, ACK36945.1), *MdXET2* (*Malus domestica*, AAN07898.1), *LcXET3* (*Litchi chinensis*, ABK30789.1), *VvXET1* (*Vitis vinifera*, AAK81880.1), *AtXET* (*Arabidopsis thaliana*, CAA63553.1), *BjXTH3* (*Brassica juncea*, AEX07607.1), *TaXTH1* (*Triticum aestivum*, AAT94293.1) and *ZaXTH1* (*Zea mays*, AAC49011.1).

**Fig 7:**
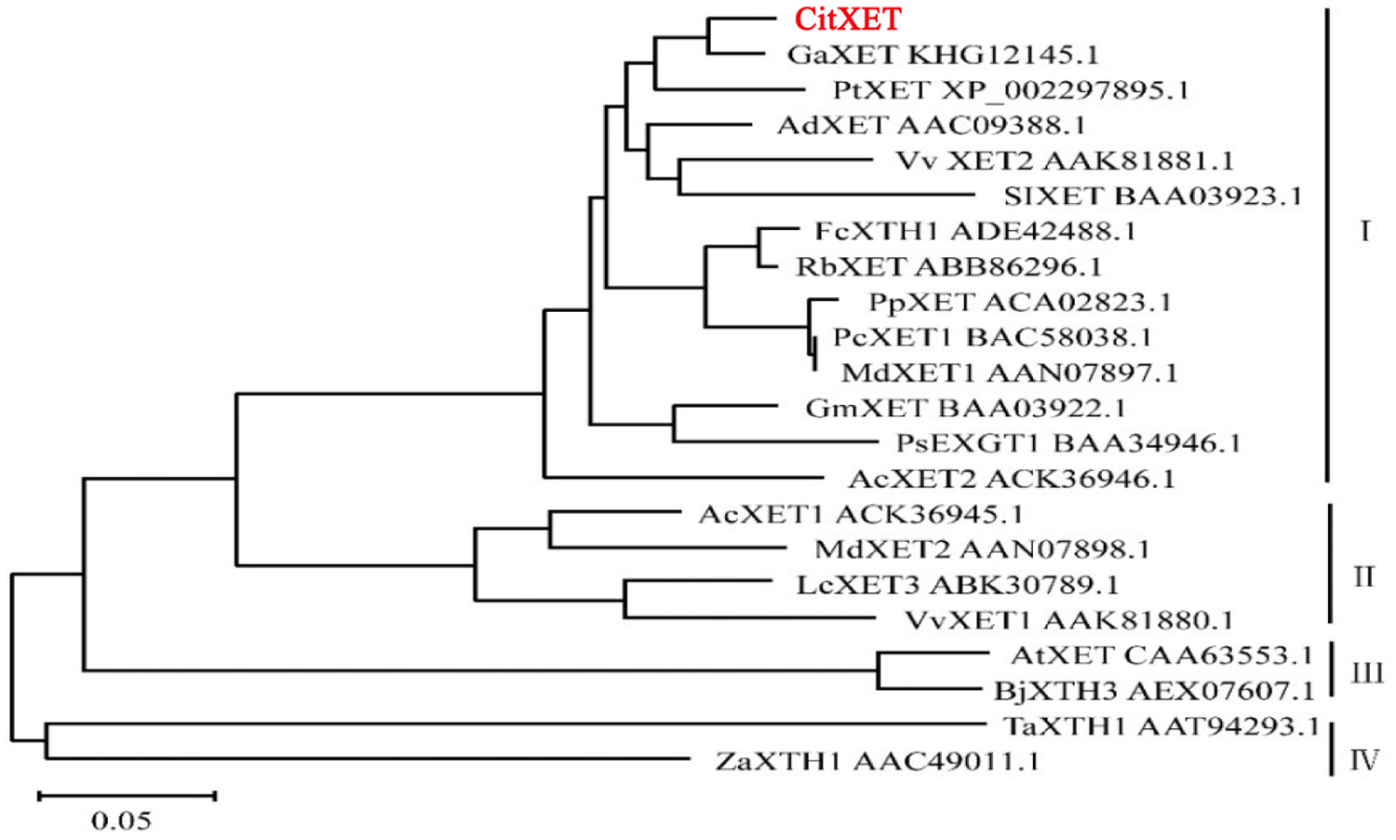
Phylogenetic tree of XETs from various plant species. The tree was generated using the neighbor-joining method of the MEGA 7.0.12 program. *GaXET* (*Gossypium arboretum*, KHG12145.1), *PtXET* (*Populus trichocarpa*, XP-002297895.1), *AdXET* (*Actinidia deliciosa*, AAC09388.1), *VvXET2* (*Vitis vinifera*, AAK81881.1), *SIXET* (*Solanum lycopersicum*, BAA03923.1), *FcXTH1* (*Fragaria chiloensis*, ADE42488.1), *RbXET* (*Rosa x borboniana*, ABB86296.1), *PpXET* (*Pyrus pyrifolia*, ACA02823.1), *PcXET1* (*Pyrus communis*, BAC58038.1), *MdXET1* (*Malus domestica*, AAN07897.1), *GmXET* (*Glycine max*, BAA03922.1), *PsEXGT1* (*Pisum sativum*, BAA34946.1), *AcXET2* (*Annona cherimola*, ACK36946.1), *AcXET1* (*Annona cherimola*, ACK36945.1), *MdXET2* (*Malus domestica*, AAN07898.1), *LcXET3* (*Litchi chinensis*, ABK30789.1), *VvXET1* (*Vitis vinifera*, AAK81880.1), *AtXET* (*Arabidopsis thaliana*, CAA63553.1), *BjXTH3* (*Brassica juncea*, AEX07607.1), *TaXTH1* (*Triticum aestivum*, AAT94293.1) and *ZaXTH1* (*Zea mays*, AAC49011.1).

An analysis of the CitXET protein secondary structure using SOPMA revealed the enzyme consists of four main structures (i.e., 21% α-helix, 30.72% extended strand, 9.09% β-turn, and 39.18% random curl) (Fig 5). The deduced amino acid sequence was compared with the XET protein sequences from other plants. Additionally, ESPript was used for homology modeling (Fig 6). The CitXET sequence and secondary structures were highly homologous to those of other plants. To investigate the evolutionary relationships between CitXET and the XETs of other plant species, we constructed a phylogenetic tree using the protein sequences for known plant XET sequences in the GenBank database (Fig 7). The phylogenetic tree was divided into two evolutionary branches. *TaXTH1* (*Triticum aestivum*, AAT94293.1) and *ZaXTH1* (*Zea mays*, AAC49011.1) clustered into one evolutionary branch (IV), while genes from the dicotyledonous plants were grouped together to form another main branch. *CitXET*, *GaXET* (*Gossypium arboreum*, KHG12145.1), and *PtXET* (*Populus trichocarpa*, XP-002297895.1) clustered together in branch I, suggesting these were the most closely related proteins.

**Fig 8:**
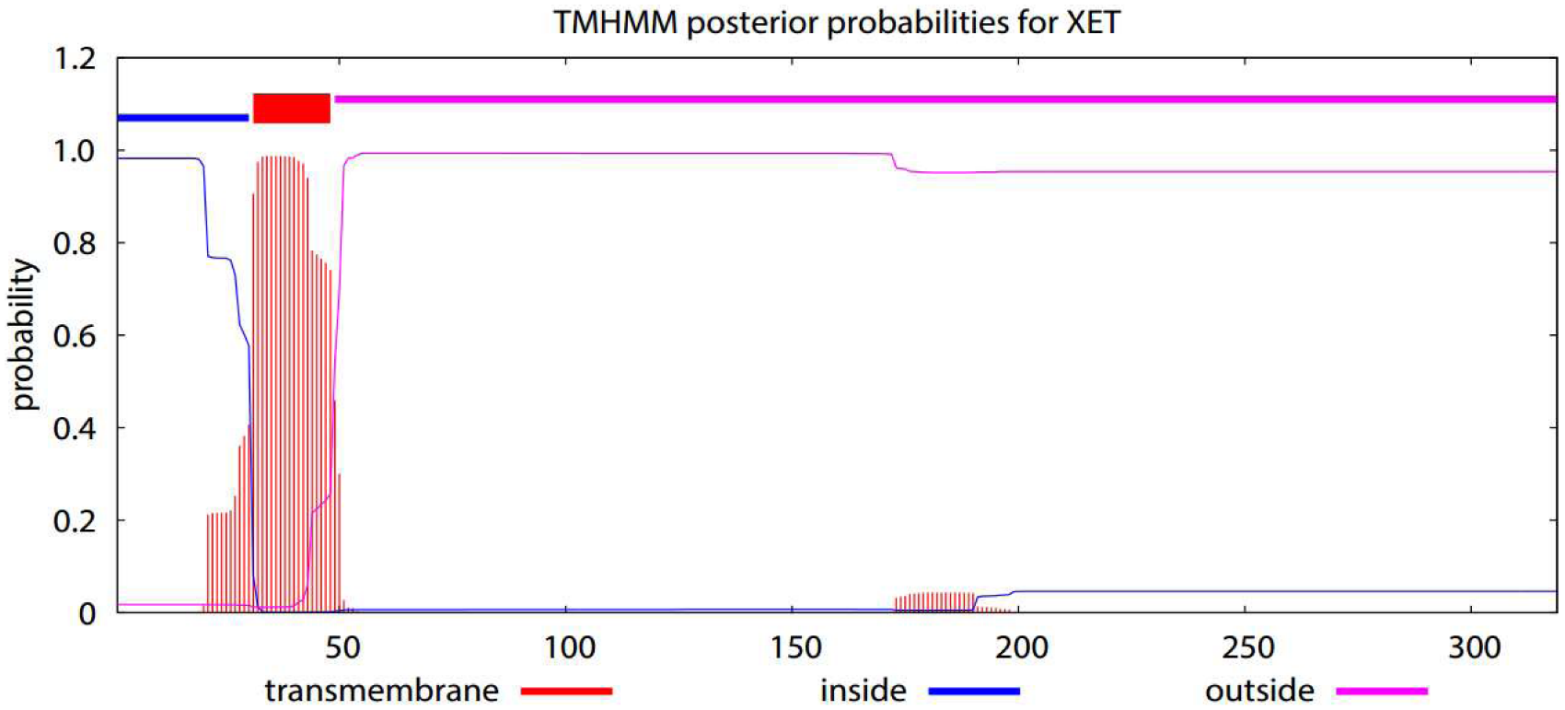
Predicted *Cit*XET protein transmembrane structures.

Using the NCBI Conserved Domains Database (NCBI-CDD), InterPro, and ScanProsite, we determined that CitXET carried the GH16 domain. The CitXET protein sequence also contained the consensus signature motif conserved among GH16 proteins (i.e., -EIDFEFLGNRT-). We used the NPS@ web server and ProScan to predict the active site of the *Huangguogan* XET protein. The results indicated that CitXET consisted of one GH16 active site, one N-glycosylation site, two protein kinase C phosphorylation sites, one casein kinase II phosphorylation site, one tyrosine kinase phosphorylation site, four N-myristoylation sites, and one amidation site (Table 2). The TMHMM server predicted that the CitXET protein had only one transmembrane structure (Fig 8). Using SignalP to identify the signal peptide revealed that the CitXET protein likely lacked a signal peptide.

**Table 2:** Predicted CitXET active sites.

Active sites	Access number	Motif	Site and Sequence
GH16 active sites	PS01034	E-[LIV]-D-[LIVF]-x(0,1)-E-x(2)-[GQ]-[KRNF]-x-[PSTA]	132 to142 EIDFEFLGNRT
N-glycosylation site	PS00001	N-{P-[ST]-{P}	140 to143 NRTG
Protein kinase C phosphorylation site	PS00005	[ST]-x-[RK]	245 to 247 SYK, 301 to 303 TDR
Casein kinase II phosphorylation site	PS00006	[ST]-x(2)-[DE]	128 to131 SEHD
Tyrosine kinase phosphorylation site	PS00007	[RK]-x(2,3)-[DE]-x(2,3)-Y	157 to 165 KGDREQRIY
N-myristoylation site	PS00008	G-{EDRKHPFYW}-x(2)-[STAGCN]-{P}	116 to 121 GSVTAF, 139 to 144 GNRTGQ, 231 to 236 GLEKTD, 253 to 258 GCEASV
Amidation site	PS00009	x-G-[RK]-[RK]	266 to 269 QGKR

**Fig 9:**
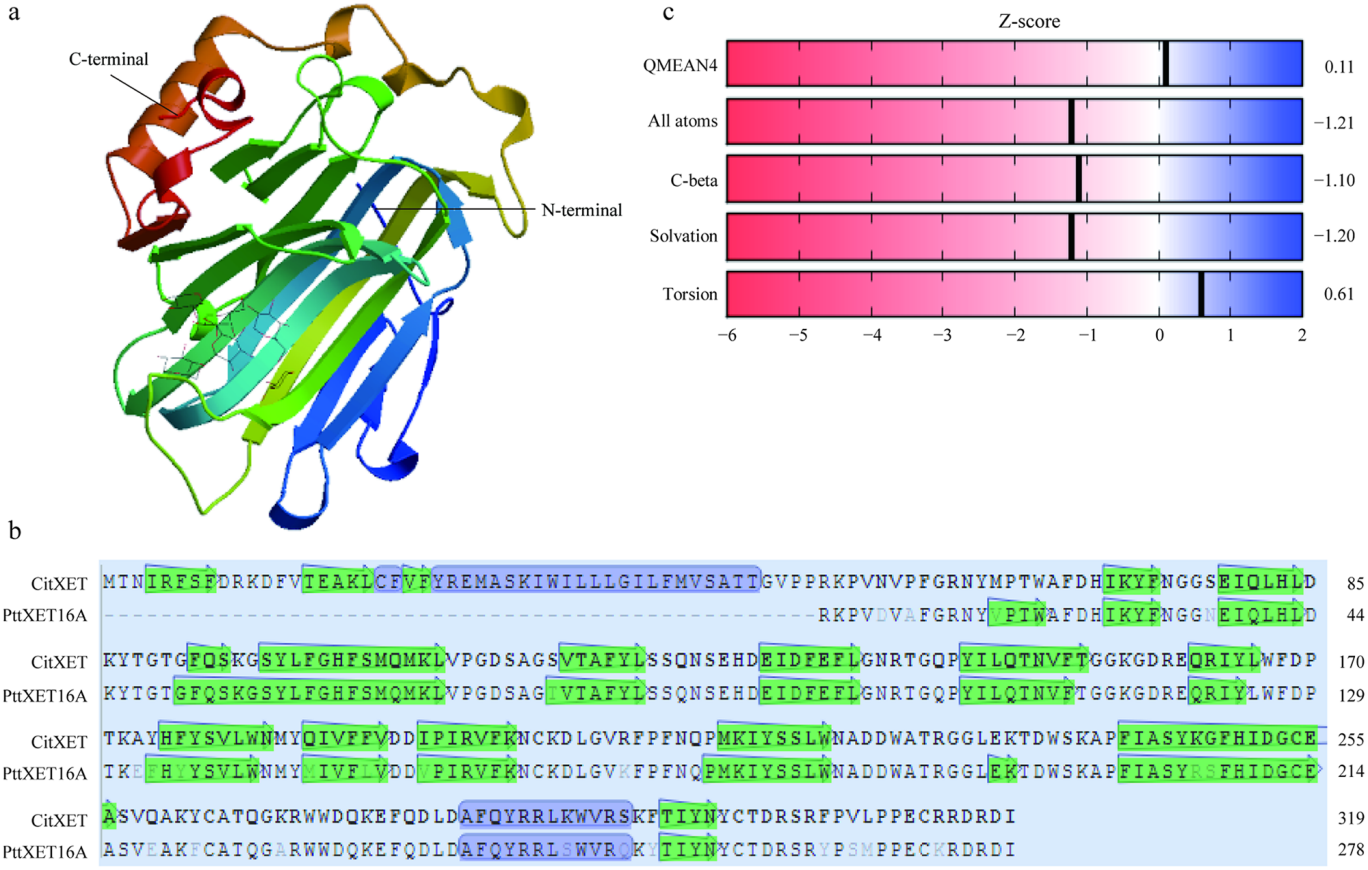
Features of the CitXET molecular structure. **a**: CitXET tertiary structures, N- to C-terminals are color-coded blue to red; **b**: Model-Template Alignment of CitXET and PttXET16A; **c**: QMEAN analysis.

The SWISS-MODEL server was used to predict the tertiary structure of CitXET based on known crystal structures of homologous proteins. The model was refined to a resolution of 1.8 Å, oligo-state was monomer, and coverage was 0.84. According to the prediction of the tertiary structure of CitXET, there were two ligands, BGC-BGC-BGC-XYS: SUGAR (4-MER) and XYS-GAL: SUGAR (2-MER), respectively. The active site residues E136, Q149, N151, E161, R163, D225, W226, and G230 were included in BGC-BGC-BGC-XYS, and D159, E161, R163, Y297, and R305 in XYS-GAL. Results regarding tertiary structure indicated that the CitXET protein was similar to other family GH16 enzymes with β-jellyroll–type structure (Fig 9a), especially PttXET16A (PDB ID: 1UMZ_A). The highest scoring (Seq identity: 91.08) and validated model for CitXET that exhibited the greatest amino acid sequence identity with the crystal structure was the protein of *Populus tremula* PttXET16A (Fig 9b). However, a notable structural feature arised because of an insertion of 41 residued at the N-terminus of CitXET, forming α-helix and β-sheet in the molecule (Figs 5, 9a and 9b). QMEAN analysis was also used to evaluate and validate the model, the QMEAN4 score was 0.11 (between 0 and 1), all atoms (−1.21), C-beta interactions (−1.10), solvation (−1.20) and torsion (0.61), which showed a good quality of the model (Fig 9c).

## Discussion

The XTHs catalyze reactions affect cell wall xyloglucans and xylans [9]. Additionally, XET activity is an important part of an ancient machinery that regulates cell wall modifications, and is common among all major groups of green plants [10], including all vascular plants [44]. To the best of our knowledge, there is limited information regarding the effects of XET on the elongation of plant roots and shoots, especially during seedling etiolation. Specific XET activity was detected in *Huangguogan* seedlings. Furthermore, we revealed a correlation between root and shoot elongation and changes in XET activity. We observed that XET activity was specific to elongation, which is consistent with the results of a previous study on liverworts [45].

Our data regarding XET activity and *CitXET* temporal expression patterns during etiolation indicated that there was a gradual increase in *CitXET* gene expression, especially between days 10 and 20, which coincided with the period when the seedling roots and shoots were rapidly growing. These findings confirmed that *CitXET* affected the etiolation of *Huangguogan* seedlings. Additionally, we detected relatively low and high *CitXET* gene expression levels in the shoots and root sub-apical regions of etiolated seedlings, respectively. The XET activities of root sub-apical region were higher than those of shoots during seedling etiolation. These implied that the higher *CitXET* expression and XET activity, the longer roots and shoots length (Table 1, Figs 2 and 3).

The XETs are members of the GH16 family, and are encoded by multigene families. Generally, the XTHs can be divided into three or four subgroups, and those belonging to classes I, II, and IIIB exhibit XET activity [36,46]. Several members of the *XET* gene family have been cloned and identified in many fruit trees. For example, three litchi, three longan, and four pear *XET* genes are available in the GenBank database. In this study, we used a homologous cloning method to isolate the complete coding sequence of the *Huangguogan XET* gene (i.e., *CitXET*), which encodes 319 amino acids (Fig 4). Based on comparisons with *XET* sequences from other plant species as well as the constructed phylogenetic tree, we determined that the N-terminal of CitXET was highly conservative. Additionally, there was a high degree of homology among the *XET* sequences from various plant species. The CitXET active site was identical to the functional site (-DEIDFEFLG-) of most GH16 XTHs (Fig 4), suggesting the catalytic domain was highly conservative. Similar results were reported by Nishitani *e*t al. [18] and Henrissat *et* al. [47]. The CitXET amino acid sequence in the catalytic domain and in the following potential N-glycosylation site [i.e., N-{P}-[ST]-{P} (access number: PS00001)] [48] (Fig 4 and Table 2) was highly homologous to sequences from other known XETs (Fig 6). *CitXET*, *GaXET* (*G*. *arboreum*, KHG12145.1), and *PtXET* (*P*. *trichocarpa*, XP-002297895.1) were clustered together in branch I, implying a close relationship among these genes (Fig 7).

## Conclusions

Our results indicate that the degree of etiolation affects the XET activity and *CitXET* expression patterns of *Huangguogan* seedlings. Furthermore, *CitXET* is vital for root and shoot growth in etiolated seedlings. The 960-bp *CitXET* coding sequence encodes a protein consisting of 319 amino acids. CitXET belongs to GH16, based on analyses using the NCBI-CDD, InterPro, and ScanProsite, and the protein has only one transmembrane structure. Our data regarding the XET-related activity and expression patterns in etiolated *Huangguogan* seedlings may be relevant to future studies on the root and shoot elongation of etiolated seedling. These studies should focus on biochemical and structural characterizations. A more thorough understanding of the effects of *CitXET* expression patterns and XET activities on root and shoot development may expand our knowledge regarding the role of XTHs during seedling etiolation.

## Supporting information

S1 Fig Agarose gel electrophoresis results for PCR-amplified *CitXET*. The band in lanes 1 and 2 in corresponds to the amplified *CitXET* gene. M: molecular weight standard (Marker III).
(TIF)
