# Supplementary figures and images for: Variability in CitXET expression and XET activity in Citrus cultivar Huangguogan seedlings with differed degrees of etiolation
Source: PLoS One. 2017 Jun 15;12(6):e0178973. doi: 10.1371/journal.pone.0178973 (PMC5472283; doi:10.1371/journal.pone.0178973)

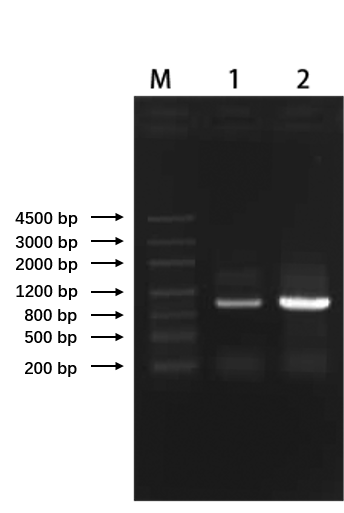

Supplement: S1 Fig — The band in lanes 1 and 2 in corresponds to the amplified CitXET gene. M: molecular weight standard (Marker III). (TIF) [file pone.0178973.s001.tif]
